# Epigenetic Clocks and EpiScore for Preventive Medicine: Risk Stratification and Intervention Models for Age-Related Diseases

**DOI:** 10.3390/jcm14103604

**Published:** 2025-05-21

**Authors:** Hidekazu Yamada

**Affiliations:** Kindai University Anti-Aging Center, 3 Chome-5-25 Hoji, Higashiosaka 577-0805, Osaka, Japan; yamadahi@med.kindai.ac.jp

**Keywords:** epigenetic clock, EpiScore, aging, preventive medicine, biological age, public health, multi-omics

## Abstract

Aging is the primary risk factor for chronic diseases such as cardiovascular disease, cancer, and dementia. However, chronological age alone fails to capture individual variability in aging trajectories and disease susceptibility. Recent advances in epigenetic clocks—DNA methylation-based models that estimate biological age—have opened new possibilities for personalized and preventive medicine. This review explores the clinical potential of epigenetic clocks and EpiScores, composite biomarkers that predict health risks and physiological status. We present a comparative evaluation of widely used epigenetic clocks, including Horvath, GrimAge, PhenoAge, and DunedinPACE, and summarize their predictive performance for mortality, cognitive decline, and cardiovascular outcomes. EpiScores linked to inflammation, glycemic control, and immunosenescence are highlighted as tools for stratified risk assessment. When integrated with multi-omics data and electronic health records, these measures enhance the precision of population health management. Special emphasis is placed on applications in longevity clinics and anti-aging clinics, community-based care, and national health checkup systems. We also explore global standardization efforts and ethical considerations, as well as Japan’s unique initiatives—including the “Aging Measurement” project at the Osaka-Kansai Expo 2025. Furthermore, we propose the development of a Global Health and Aging Index that integrates the biological, functional, and subjective dimensions of aging, aligned with the WHO concept of Intrinsic Capacity. In conclusion, epigenetic clocks and EpiScores represent transformative tools for shifting from reactive treatment to proactive health optimization, and from chronological to biological metrics in aging science and public health policy.

## 1. Introduction

Aging is the greatest risk factor for chronic diseases such as diabetes, cardiovascular disease, and dementia. Traditionally, chronological age has been used to evaluate aging, but this does not adequately reflect the significant individual variations in the progression of aging. In recent years, the development of biomarkers that objectively capture the biological processes of aging has gained attention. Among these, epigenetic clocks based on DNA methylation patterns have emerged as a promising tool.

Epigenetic clocks quantify biological age and provide a dynamic assessment of “accelerated aging” or “rejuvenation”, showing potential for early detection, preventive interventions, and applications in population health strategies.

This review examines the potential of epigenetic clocks and EpiScores as practical biomarkers in clinical settings. We will explore how these tools can be utilized for disease prediction, response to lifestyle interventions, and integration into routine healthcare. Furthermore, we will discuss their contribution to enhancing health screening systems, supporting behavioral changes, and realizing precision public health aimed at extending healthy life expectancy.

## 2. Evolution of Epigenetic Clocks and Clinical Possibilities

Epigenetic clocks are models that estimate biological age by analyzing age-related DNA methylation changes at specific CpG sites in the genome. These models have evolved over generations, increasing their relevance to clinical and public health outcomes (Table 1).

### 2.1. First-Generation Clocks: Chronological Age Estimation Models

Early epigenetic clocks developed by Horvath [1] and Hannum [2] aimed to predict chronological age with high accuracy using Elastic Net regression. The Horvath clock, based on 353 CpG sites, is characterized by its applicability across multiple tissues. However, these models were not optimized for predicting health outcomes and have limitations in their clinical application.

### 2.2. Second-Generation Clocks: Predicting Healthy Lifespan and Mortality Risk

To overcome these limitations, second-generation clocks such as PhenoAge [3] and GrimAge [4] were developed. PhenoAge incorporates clinical biomarkers like albumin, creatinine, and CRP, while GrimAge includes methylation proxy indicators of plasma proteins and smoking history. These models show a higher correlation with disease onset and mortality risk and have been shown to be effective in stratifying cardiovascular disease, type 2 diabetes, cancer, and dementia [5].

### 2.3. Third- and Fourth-Generation Clocks: Evaluating Aging Rate and Functional Aging

A newer model, DunedinPACE [6], aims to quantify the “pace of aging” rather than biological age. Based on longitudinal cohort studies, it is designed to measure accumulated functional decline.

Fourth-generation approaches are advancing with the integration of tissue-specific methylation profiles and multi-omics information, expected to deepen our understanding of disease specificity and causal relationships.

For example, Polycomb-associated clocks [7] utilize increased DNA methylation at PRC2 (Polycomb Repressive Complex 2) target sites, reflecting tissue-specific aging processes and sensitivity to epigenetic reprogramming. These clocks are mechanistically linked to important biological processes associated with aging, such as tumor formation and the suppression of differentiation.

### 2.4. Epigenetic Age Acceleration and Epidemiological Significance

The difference between epigenetic age and chronological age (Epigenetic Age Acceleration = EAA) has gained attention as a predictor of health outcomes. Large-cohort studies such as the Framingham Heart Study and Generation Scotland have reported associations between EAA and outcomes such as frailty, metabolic syndrome, cardiovascular events, mortality, and cognitive impairment [8,9,10].

Each clock reflects different biological pathways. For example, GrimAge [11] is associated with inflammation and hematopoietic aging, PhenoAge [12] with immune aging and metabolic stress, and DunedinPACE [13] with the accumulation of functional burden. This diversity allows researchers and clinicians to select clocks based on their specific objectives.

### 2.5. Methodological Challenges and Limitations

While epigenetic clocks are useful, they also face several technical challenges. These include technical variations between platforms such as Illumina 450k and EPIC arrays, batch effects, and confounding due to differences in cell type composition.

Additionally, many models have been trained on data from Western populations, presenting challenges in terms of their generalizability to different ethnic groups. For their future widespread adoption, the development of ethnicity-specific models and standardized analytical methods is essential.

## 3. Comparative Evaluation of Epigenetic Clocks

Diverse types of DNA methylation-based epigenetic clocks have been developed for estimating biological age, predicting health outcomes, and evaluating age-related interventions. These clocks differ in their training objectives, applicable tissues, predictive accuracy, and responsiveness to environmental and lifestyle factors. This chapter compares representative epigenetic clocks—Horvath Clock, Hannum Clock, PhenoAge, GrimAge, and DunedinPACE—summarizing their advantages and limitations in Table 1.

### 3.1. First-Generation Clocks: Chronological Age Prediction Type

The Horvath Clock (2013) was the first multi-tissue compatible epigenetic clock, aiming to predict chronological age across various human tissues [14]. Its strengths lie in its cross-tissue applicability and high correlation with chronological age, making it useful as a baseline for biological age estimation. The Hannum Clock was also trained to predict chronological age with high accuracy using blood samples [15]. However, both have limited power in predicting functional decline and disease onset, presenting limitations for clinical risk assessment.

### 3.2. Second-Generation Clocks: Phenotype and Mortality Risk Prediction Type

PhenoAge was developed by Levine et al. and trained based on clinical biomarkers associated with morbidity and mortality [15]. It excels in evaluating functional aging and is also associated with frailty, inflammation, and immune decline. GrimAge incorporates methylation proxy indicators of plasma proteins (GDF-15, PAI-1, leptin, etc.) and smoking history [16], demonstrating an excellent performance in predicting time to death, cardiovascular events, and cancer onset.

### 3.3. Third-Generation Clocks: Evaluating Aging Rate (Pace)

The DunedinPACE clock was developed from longitudinal data based on a birth cohort and reflects the speed of aging rather than accumulated age [17]. It is designed to capture changes that occur over short periods due to lifestyle interventions or treatments and is widely used in recent intervention studies, clinical trials, and behavioral aging research.

### 3.4. Applications in Clinical and Research Settings

**Figure 1:**
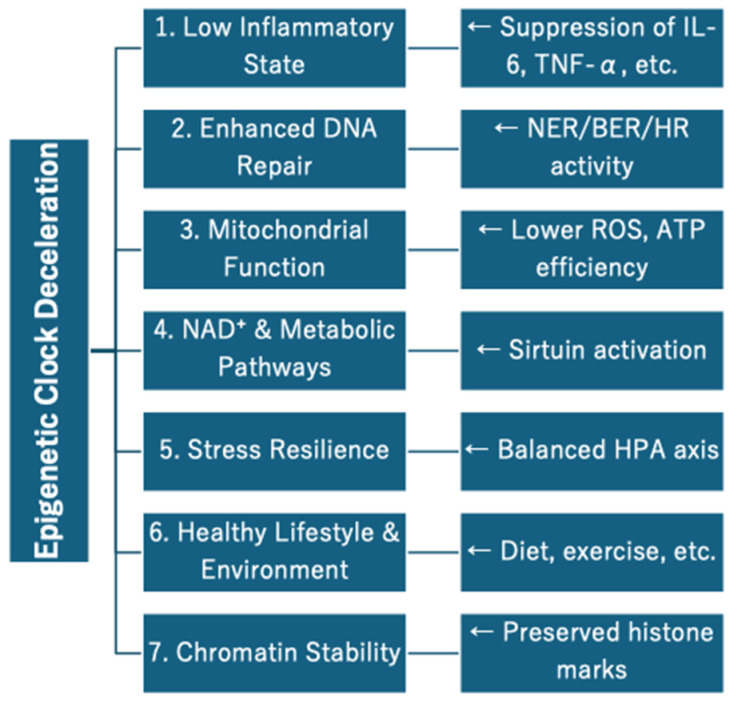
Biological pathways associated with aging, as reflected by different epigenetic clocks.

Each clock reflects different biological pathways. GrimAge is associated with the blood hematopoietic system and inflammation, PhenoAge with immune aging and metabolic stress, and DunedinPACE with functional decline and multi-disease comorbidity. Which clock to use should be selected according to its purpose, whether for individual health monitoring, population-based risk stratification, or the evaluation of anti-aging interventions (Figure 1).

## 4. Risk Stratification with EpiScore and Multi-Omics

In addition to epigenetic clocks, synthetic indicators based on DNA methylation patterns called EpiScores have been developed and associated with specific proteins and clinical phenotypes [18]. These scores provide information focused on specific pathways related to aging and health, which is effective in improving the precision of risk stratification in the elderly. Furthermore, a comprehensive assessment is possible through integrating multi-omics data such as proteomics, metabolomics, and transcriptomics, contributing to the identification of individuals at high risk for disease [19].

### 4.1. Definition and Development of EpiScores

EpiScores are constructed using machine learning on large-scale epidemiological datasets, aiming to predict protein concentrations, cytokines, and clinical biomarkers from DNA methylation data. Unlike clocks aimed at age estimation, EpiScores focus on predicting disease states, physiological characteristics, and environmental exposure. They have been validated in several cohorts, with high predictive accuracy reported [19].

### 4.2. Disease-Specific Applications

- **Inflammation**: EpiScores for inflammatory markers such as IL-6, TNF-α, and CRP are useful for assessing chronic inflammatory burden and predicting the risk of metabolic syndrome and cardiovascular diseases [20].
- **Type 2 Diabetes**: Methylation-based scores correlated with HbA1c and insulin resistance help in the early detection of prediabetes and metabolic risks [14].
- **Cardiovascular Disease**: Proxy markers included in GrimAge, such as GDF-15, PAI-1, and leptin, are strongly associated with atherosclerosis, heart failure, and mortality risk [19].
- **Immune Aging (Immunosenescence)**: EpiScores related to an immune function decline contribute to infection risk stratification and vaccine response prediction in the elderly [15].

### 4.3. Integration with Multi-Omics

The integration of EpiScores with transcriptomics, proteomics, and metabolomics enables the elucidation of disease mechanisms and the identification of intervention targets. For instance, combining epigenetic clocks with proteomic markers has been reported to improve prediction accuracy for frailty, cognitive decline, and multi-disease comorbidity [19,21].

### 4.4. Individual and Regional Applications of EpiScores 

EpiScores visualize “hidden risks” before clinical symptoms appear, aiding in the personalization of behavioral changes and preventive measures. In regional public health, they can evaluate the accumulation of biological stress in populations and optimize resource allocation and intervention-planning. They are expected to be integrated with mobile apps and electronic health records (EHR) for use in daily health monitoring and feedback systems.

## 5. Disease-Specific Applications: Dementia, Cancer, and Cardiovascular Disease

Epigenetic clocks and EpiScores are increasingly important in predicting and monitoring age-related diseases. Aging is the greatest risk factor for many chronic diseases, and DNA methylation-based biomarkers contribute significantly to understanding disease onset, disease progression, and treatment response. This chapter introduces applications in three major areas: dementia, cancer, and cardiovascular disease.

### 5.1. Dementia and Cognitive Decline

Multiple studies have shown associations between epigenetic age acceleration and cognitive impairment. Individuals with a high GrimAge or PhenoAge tend to show memory decline, executive function impairment, and an early decline in age-related cognitive function [16,22]. These also correlate with brain atrophy and neuroinflammation, characteristics of Alzheimer’s disease.

Recently, the development of brain-specific methylation clocks has advanced, offering hope for more tissue-specific predictions. Furthermore, integration with polygenic risk scores and neuroimaging (e.g., hippocampal volume and tau PET) may enable the early detection of preclinical Alzheimer’s disease [23].

### 5.2. Cancer

Cancer risk increases with age, and epigenetic abnormalities are central elements in tumor formation. The acceleration of GrimAge is associated with the onset of lung, breast, colorectal, and prostate cancers [16,24]. These methylation changes reflect the processes of tumor formation, such as chronic inflammation, immune evasion, genomic instability, and cellular senescence.

Additionally, EpiScores specialized in pathways such as p53 signaling, DNA damage response, and angiogenesis enable the more precise stratification of cancer risk. Furthermore, their combination with circulating tumor DNA (ctDNA) and exosome analysis opens new possibilities for minimally invasive cancer diagnosis and treatment monitoring.

### 5.3. Cardiovascular Disease (CVD)

Epigenetic clocks show the strongest and most consistent evidence of an association with cardiovascular disease (CVD). GrimAge and PhenoAge strongly correlate with atherosclerosis, coronary artery calcification, left ventricular hypertrophy, and risks of myocardial infarction and heart failure [16,25].

In particular, EpiScores based on biomarkers such as leptin, GDF-15, and PAI-1 enhance the prediction accuracy of cardiovascular events beyond traditional risk models such as the Framingham score. This greatly contributes to preventive medicine by enabling early intervention at asymptomatic stages.

In countries like Japan that have advanced health screening systems yet still have cardiovascular disease as a major cause of death, incorporating biological aging markers into health examinations would strengthen primary prevention and risk stratification.

## 6. Integration into Preventive and Personalized Medicine

Incorporating epigenetic clocks and EpiScores into preventive medicine is a promising approach that fundamentally changes how we assess and manage the risks of aging and age-related diseases. In particular, conventional health examination models that rely on chronological age or uniform risk criteria may not adequately reflect an individual’s biological resilience or vulnerability. DNA methylation-based biomarkers can be integrated into routine clinical care as more dynamic and personalized indicators of health status.

### 6.1. Limitations and Challenges

Despite their growing utility, epigenetic clocks and EpiScores face several limitations. These include inter-platform variability (e.g., 450K vs. EPIC 850K arrays), poor reproducibility across laboratories, and tissue-specific differences in accuracy. Additionally, clocks trained in predominantly European populations may underperform in non-European groups. EpiScores offer disease-specific insights but are not yet widely validated or cost-effective. Integration into clinical systems remains limited due to a lack of standardization and regulatory oversight. Comparisons with protein biomarkers and polygenic risk scores (PRS) show mixed results, suggesting the need for multi-modal approaches. Broader efforts in international standardization, such as ISO-level guidelines and ethnicity-specific model development, are ongoing and essential for widespread adoption [26].

### 6.2. Strengthening Health Screening Systems and Improving Risk Communication

In Japan, specific health examinations are mandatory for those aged 40–74, aiming to achieve the early detection of metabolic syndrome and to prevent lifestyle-related diseases. However, this screening system mainly relies on standard indicators, such as BMI, blood pressure, and cholesterol, making it difficult to capture early biological changes associated with aging.

Incorporating “biological age” into such systems greatly improves the accuracy of risk assessment. For example, a 50-year-old with a biological age of 60 may be judged to need stronger intervention than someone with a biological age of 45. Feedback like “your biological age is 10 years older than your actual age” is intuitively easy to understand and serves as powerful motivation, especially for asymptomatic individuals.

Healthcare professionals such as public health nurses, primary care physicians, and dietitians can use this information to personalize lifestyle improvement guidance. In fact, counseling using biological age has been reported to improve patient engagement and intervention continuation rates.

### 6.3. Monitoring the Effects of Lifestyle Interventions

One of the most promising applications of epigenetic clocks is evaluating intervention effects. Multiple studies have shown that interventions in diet, exercise, sleep, and stress management can delay or even rejuvenate biological age [25,27,28]. For instance, caloric restriction and aerobic exercise have been confirmed to lower epigenetic age within months, as measured by GrimAge and DunedinPACE.

By measuring biological age before and after interventions, healthcare providers and patients can objectively confirm the effects of interventions. This promotes behavioral change and enables the redesign of personalized strategies. The longitudinal tracking of aging indicators also serves as a guide to determine whether intensification or adjustment of the intervention is necessary [29].

### 6.4. Prospects for Personalized Preventive Medicine

With the decreasing costs of methylation testing and increasing test availability, the feasibility of implementing personalized preventive medicine continues to expand. Integration with electronic health records (EHR), wearable devices, and mobile health apps enables the continuous monitoring of biological age and real-time feedback.

Ultimately, combining epigenetic clocks with EpiScores and multi-omics information (microbiome, transcriptome, metabolome, etc.) will lead to more comprehensive individual profiling. Such efforts support the transition from conventional “medicine that treats after symptoms appear” to a “proactive approach to maintain biological function”. This is an essential shift, especially for super-aged societies like Japan.

## 7. Applications in Longevity Clinics and Community Care

Epigenetic clocks were initially developed for research purposes but are now being applied in clinical settings, particularly in longevity clinics and anti-aging clinics. These facilities provide personalized strategies to optimize aging progression, prevent age-related diseases, and improve quality of life. There is also growing interest in population health management using biological age measurements in public health and community comprehensive care settings.

### 7.1. Utilization in Longevity and Anti-Aging Clinics

Longevity clinics in America, Japan, Singapore, UAE, and other countries have introduced biological age assessments using epigenetic clocks as part of comprehensive health examinations. Patients undergo DNA methylation testing and receive individualized recommendations on diet, exercise, supplements, and regenerative medicine based on the results.

Testing companies with CLIA certification, such as TruDiagnostic and Zymo Research, contribute to ensuring clinical reliability and international comparability. In Japan, some clinics in Tokyo and Osaka have begun incorporating epigenetic clocks into standard anti-aging programs, conducting longitudinal follow-ups and measuring the effects of lifestyle interventions.

Such applications are not just privileges for the wealthy. With declining testing costs and increasing social interest, their use is spreading to wellness-oriented citizens and the working generation in their 40s to 60s.

### 7.2. Utilization for Community Comprehensive Care and Frailty Prevention

In aging societies like Japan, the significance of biological age measurement is increasing in the context of comprehensive community care and frailty prevention. For example, even elderly people who appear healthy may benefit from early intervention (resistance training, nutritional support, social participation programs, etc.) if their biological age is significantly advanced.

Additionally, biological age feedback enhances the effectiveness of the behavioral change support provided by public health nurses, dietitians, and municipal staff. Biological age is more intuitively understandable than abstract biomarkers and serves as an easily usable indicator for patients’ behavioral guidance.

### 7.3. Ethical and Implementation Challenges

Some municipalities have begun pilot projects that link biological age measurement, though not at the epigenetic clock level, with regional wellness platforms and incentive systems. For example, models in which residents can earn rewards by engaging in health behaviors linked to health point systems are being tested.

The use of epigenetic clocks is also being considered as an objective intervention criterion for frailty prevention in nursing care. This would make it possible to allocate care prevention resources more accurately.

A notable case is the Osaka-Kansai Expo 2025. The Osaka Pavilion plans to offer visitors the opportunity to visualize their biological age on the spot through non-invasive tests like grip strength, body composition balance, dental alignment, and cognitive measurements [30]. This represents an important step in spreading a new perspective in society: not “How old are you?” but “How healthily are you aging?”

### 7.4. Roadmap for Clinical Integration

**Figure 2:**
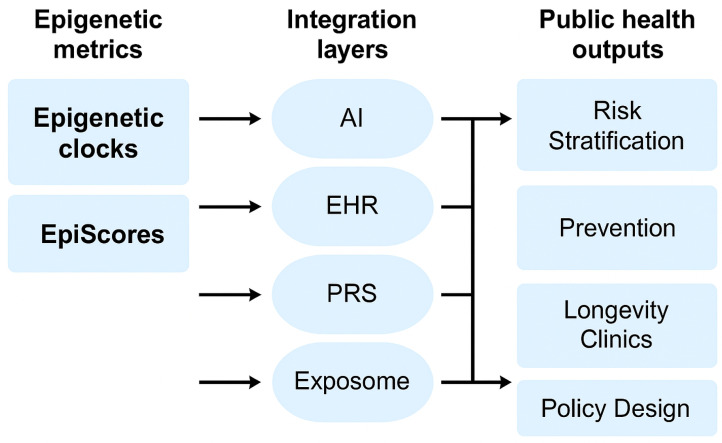
Multi-layered integration of epigenetic clocks and EpiScores with digital health infrastructure and public health outputs. This diagram illustrates how epigenetic clocks and EpiScores are connected to public health outcomes via intermediate integration layers, including artificial intelligence (AI), electronic health records (EHR), polygenic risk scores (PRS), and exposome data.

Figure 2 provides a synthesized framework illustrating how EpiScores and epigenetic clocks can be integrated into clinical practice and public health systems, aligning with the discussed implementation strategies.

## 8. Digital Health and Real-Time Feedback (Revised Version)

**Figure 3:**
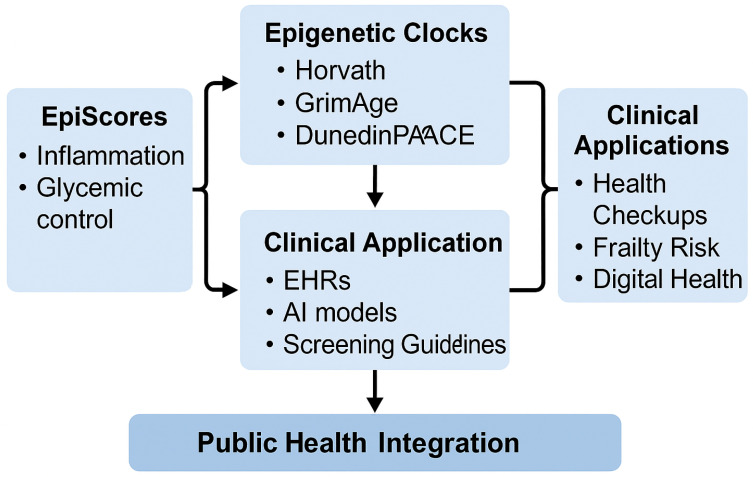
Multi-layered framework connecting epigenetic clocks, EpiScores, and AI-enabled public health integration. As shown in Figure 3, we propose an integrated framework in which EpiScores complement epigenetic clocks to enhance their clinical applicability in screening, monitoring, and preventive care pathways. This model emphasizes modular risk profiling, combining biological aging indicators with disease-specific methylation signatures.

A conceptual illustration of how EpiScores and epigenetic clocks are integrated with AI infrastructure and public health systems is presented in Figure 3.

Furthermore, the concept of “Intrinsic Capacity (IC)” proposed by the World Health Organization (WHO) focuses on the positive aspects of aging, providing a framework that comprehensively captures physical, cognitive, and psychological functions [25]. By comprehensively evaluating subjective well-being (e.g., life satisfaction, self-efficacy, and happiness) in addition to epigenetic indicators such as biological age and EpiScores, truly personalized aging support and preventive medicine can be realized.

Additionally, epigenetic clocks and EpiScores can dramatically enhance their interpretative power and predictive accuracy when combined with multi-omics data such as transcriptomics, proteomics, and metabolomics. Such integrated indicators capture the progression of aging more multidimensionally and are being utilized for the early risk assessment of diseases and evaluation of lifestyle intervention effects.

In the future, visualizing and tracking individual “health stories” from both objective multi-omics data and subjective health perceptions will be the key to next-generation digital preventive medicine.

### 8.1. Integration with Electronic Health Records (EHR)

As electronic health record (EHR) systems expand their functionality to handle genomic, epigenomic, and multi-omics data, the introduction of epigenetic age is reaching a realistic stage. Biological age can be incorporated into daily clinical practice as a new “next-generation vital sign”, in addition to conventional vital signs such as blood pressure, cholesterol, and BMI.

For example, patients with advanced biological age despite normal test values are identified as candidates for early intervention against hidden risks. Conversely, for individuals showing healthy aging, examination frequency and intervention levels can be flexibly adjusted.

Additionally, by adding multi-omics indicators (e.g., gut microbiota, blood metabolites, and inflammation markers), more precise health profiling and predictive models are being developed within EHRs.

### 8.2. Mobile Health and Personalized Coaching

Mobile health (mHealth) technology and wearable devices provide a new means to visualize the interface between biological age and daily life in real-time. Individuals can import their DNA methylation test results into apps and receive dynamic reports and behavioral suggestions.

By providing feedback on the relationship between daily life factors such as physical activity, sleep, stress, nutrition, and biological age or EpiScores, users can “visualize” the impact of their lifestyle habits on aging. This serves as an extremely powerful motivation for behavioral change.

Furthermore, more comprehensive and self-directed health coaching becomes possible by combining self-assessments based on the Intrinsic Capacity framework (e.g., mood, vitality, and concentration).

### 8.3. Challenges and Future Prospects

Several challenges exist in the social implementation of such systems. These include the standardization of measurement protocols, interoperability between platforms, legal and ethical regulations, and the establishment of privacy protection. An intuitive and interpretable feedback design for both healthcare providers and patients is also necessary.

Nevertheless, as precision medicine and public health converge, biological age indicators will become central to dynamic and predictive health management through the coordination of multi-omics and AI technology.

In the future, such indicators will function as a “comprehensive aging dashboard” integrated with Intrinsic Capacity and subjective well-being, making it realistic for individuals and society to co-design a healthy life expectancy.

## 9. Integration with AI and Exposome

The future of aging science lies at the intersection of molecular biomarkers, environmental exposure data, and advanced analytical methods. Epigenetic clocks and EpiScores, when integrated with artificial intelligence (AI) and the exposome (lifetime environmental exposures), open new paths to precision public health and personalized aging management.

### 9.1. Biological Aging Modeling with AI

AI possesses the ability to process high-dimensional, multi-layered data such as DNA methylation, transcriptomics, proteomics, metabolomics, and life logs. Machine learning algorithms elucidate complex relationships between specific epigenetic signatures and disease risk, aging rate, and health outcomes.

For example, deep learning models can automatically estimate biological age from raw methylation β values, enabling risk stratification in populations. Based on longitudinal data, predictions of aging trajectories and intervention simulations are also possible.

Furthermore, AI supports the construction of individual aging profiles and digital twins (in silico reflection of lifestyle and treatment effects). This enables the realization of biologically personalized and dynamically adaptive prevention strategies.

### 9.2. Exposome and Environmental Epigenetics

The exposome refers to the cumulative environmental exposures (air pollution, diet, psychological stress, chemicals, infections, and lifestyle habits) throughout life. These interact with the genome and epigenome, shaping health trajectories over decades.

Epigenetic clocks show sensitivity to many elements of the exposome, including PM2.5, noise, nutritional deficiencies, childhood adversity, and occupational exposures. Specific DNA methylation sites can serve as biomarkers for exposure to toxins and chronic stress.

EpiScores reflecting inflammation, oxidative stress, and metabolic abnormalities can also function as intermediate phenotypes between the exposome and disease. The integration of exposome and epigenetics enables an understanding of the environmental origins of aging and identification of modifiable risk factors.

### 9.3. Applications to Precision Public Health

Combining AI and exposome analysis with epigenetic clocks enables a transition from “one-size-fits-all” prevention to stratified population prevention. This approach allows for the early identification of high-risk individuals and the more equitable distribution of public resources.

In the future, digital platforms are expected to provide users with personalized advice based on cumulative exposure history and biological age. At the municipal and national levels, these tools can be used to understand population aging trends and health disparities, optimizing policy development.

## 10. Global Standardization and Policy Prospects

As epigenetic clocks and EpiScores progress from research stages to real-world applications, the need for their international standardization and integration into policies is rapidly increasing. Harmonizing aging biomarkers across countries, ethnicities, regulatory systems, and healthcare infrastructures is essential to ensure scientific reliability, clinical validity, and equitable access.

### 10.1. International Initiatives and Validation Trends

International organizations and foundations such as the World Health Organization (WHO), Hevolution Foundation, and XPRIZE for Healthspan are working to establish advanced frameworks that view aging as a “measurable and modifiable biological process”. While global consensus has not yet been reached, these movements are noteworthy for their focus on visualizing and preventing aging.

These efforts aim to validate epigenetic clocks across ethnic and regional differences, conduct long-term longitudinal tracking of aging interventions, and build international consortia for data-sharing.

PRC2-AgeIndex, GrimAge, and DunedinPACE have shown promising results in international validation studies, but challenges remain in terms of region-specific adjustments. The importance of local adaptation, particularly in Asia, Africa, and Latin America, is crucial to avoid widening health disparities.

### 10.2. Regulatory and Ethical Challenges

Incorporating biological age into healthcare systems brings several ethical and legal challenges, such as the following:

- Should insurance premiums vary according to biological age?
- How should biological age be utilized in workplace health assessments?
- What protective measures are needed to prevent age-based discrimination?

In response to these concerns, the international bioethics community is developing frameworks that emphasize privacy, fairness, and empowerment. Biological age information should be used to support individual care and autonomy, not to disadvantage individuals based on factors beyond their control.

Additionally, the regulatory approval of diagnostic tools based on methylation testing (such as CLIA in the US, CE-IVD in Europe, and PMDA in Japan) is also evolving to reflect the clinical significance of aging biomarkers.

### 10.3. Towards Building Global Aging Indicators

With the progression of global aging, new frameworks are needed to scientifically and equitably extend healthy life expectancy. The construction of a “Global Health and Aging Index” or “Well-being Score” that integrates biological age measurement technologies (epigenetic clocks, EpiScores), functional indicators, environmental factors, and subjective well-being assessments is attracting attention as the next stage.

Such indicators are very useful for various purposes, including the following:

- Real-time understanding of the aging status of populations in each country and region.
- Early stratification of disease onset risk and prioritization of appropriate interventions.
- Scientific and equitable allocation of healthcare, nursing care, and health resources.
- “Visualization” of health disparities due to social and economic factors.

For example, Intrinsic Capacity, proposed by WHO, is a concept that comprehensively captures the physical, mental, and social functions of the elderly and is gaining international traction. It is also consistent with the European Union’s “Healthy Aging” policy and aging response measures in Asian countries, gradually paving the way for the international standardization of comprehensive aging indicators.

In Japan, the “Visualization of Aging” project at the Osaka-Kansai Expo 2025 is attracting attention. Visitors will gain experience confronting their biological age through epigenetic clocks and well-being scores [31]. This serves as an opportunity to transform the perception of aging from “invisible” to “visible,” and then to “changeable.”

The ultimate goal is to balance evidence-based health policies and personalized preventive interventions. Building a measurable, modifiable, and equitably distributable paradigm of healthy longevity through collaboration between government, academic institutions, industry, and civil society will be an important mission in 21st-century public health.

## 11. Conclusions

The rapid evolution of epigenetic clocks and EpiScores has opened new horizons in precision medicine within aging science. These DNA methylation-based biomarkers are no longer confined to the research domain but are beginning to be applied in clinical and public health fields. This has made scalable, dynamic, and biologically meaningful tools available for predicting the risk of age-related diseases, designing interventions, and evaluating health status.

Epigenetic clocks such as GrimAge and DunedinPACE show strong correlation with morbidity and mortality predictions, while EpiScores contribute to visualizing aging pathways such as inflammation, metabolic abnormalities, and immune aging. These enable a transition from conventional symptom-based medicine to proactive and data-driven preventive medicine.

Integration into annual health examinations, community comprehensive care, mobile health applications, and AI-assisted platforms holds great potential for personalized lifestyle guidance, early intervention, and long-term risk reduction. Using biological age feedback also promises to enhance health literacy, maintain patient motivation, and promote preventive behaviors.

Internationally, efforts are advancing toward the standardization of aging biomarkers and global indicator development, with Japan—possessing both population structure challenges and technological innovation—in a position to lead the world in demonstrating applications. Particularly noteworthy are the Osaka-Kansai Expo 2025 and the development of frailty prevention networks in communities.

In the future, the fusion of epigenetic clocks, EpiScores, exposome analysis, and AI will construct comprehensive aging indices and new frameworks for health value assessments. Such a transformation will not limit biological aging to a mere clinical indicator but serve as the foundation for a new social model that measures and utilizes “health capital” at individual, healthcare system, and national levels.

Ultimately, as the regular measurement and application of biological age become widespread in clinical and public health practice, our understanding, measurement, and management of “aging” will be redefined, enabling the realization of a society that can extend not just lifespan but healthy life expectancy more equitably and proactively, based on scientific evidence.

## Figures and Tables

**Table 1:** Comparison of major epigenetic clocks.

Clock Name	Purpose	Strengths	Limitations	Main Uses
Horvath	Chronological age estimation	Multi-tissue compatible Benchmark tool	Poor disease prediction power	Biological age standard
Hannum	Chronological age estimation	High accuracy in blood samples	Limited tissue applicability	Aging research
PhenoAge	Phenotypic biomarkers	Excellent for morbidity/frailty prediction	Sensitive to clinical variations	Clinical risk prediction
GrimAge	Mortality Protein markers	High predictive power for lifespan	Susceptible to lifestyle influences	Cardiovascular cancer research
DunedinPACE	Aging rate	Sensitive to interventions Suitable for longitudinal studies	Requires special analysis	Intervention lifestyle research

## Data Availability

No new data were created or analyzed in this study. Data sharing is not applicable to this article.
